# Enhancing Single-Cell and Bulk Hi-C Data Using a Generative Transformer Model

**DOI:** 10.3390/biology14030288

**Published:** 2025-03-12

**Authors:** Ruoying Gao, Thomas N. Ferraro, Liang Chen, Shaoqiang Zhang, Yong Chen

**Affiliations:** 1 College of Computer and Information Engineering, Tianjin Normal University, Tianjin 300387, China; tjnugry@163.com (R.G.); 2210090012@stu.tjnu.edu.cn (L.C.); 2 Department of Biomedical Sciences, Cooper Medical School of Rowan University, Camden, NJ 08103, USA; ferrarot@rowan.edu; 3 Department of Biological and Biomedical Sciences, Rowan University, Glassboro, NJ 08028, USA

**Keywords:** Hi-C, scHi-C, data imputation, transformer model, deep learning

## Abstract

The 3D organization of chromatin in the nucleus plays a critical role in regulating gene expression and maintaining cellular functions in eukaryotic cells. High-throughput chromosome conformation capture (Hi-C) and its derivative technologies have been developed to map genome-wide chromatin interactions at the population and single-cell levels. However, insufficient sequencing depth and high noise levels in bulk Hi-C data, particularly in single-cell Hi-C (scHi-C) data, result in low-resolution contact matrices, thereby limiting diverse downstream computational analyses in identifying complex chromosomal organizations. To address these challenges, we developed a transformer-based deep learning model, HiCENT, to impute and enhance both scHi-C and Hi-C contact matrices. Validation experiments on large-scale bulk Hi-C and scHi-C datasets demonstrated that HiCENT achieves superior enhancement effects compared to five popular methods. When applied to real Hi-C data from the GM12878 cell line, HiCENT effectively enhanced 3D structural features at the scales of topologically associated domains and chromosomal loops. Furthermore, when applied to scHi-C data from five human cell lines, it significantly improved clustering performance, outperforming five widely used methods. The adaptability of HiCENT across different datasets and its capacity to improve the quality of chromatin interaction data will facilitate diverse downstream computational analyses in 3D genome research, single-cell studies and other large-scale omics investigations.

## Simple Summary

Understanding how DNA is organized within a cell’s nucleus is essential for studying gene activity and cellular function. Scientists use specialized sequencing technologies, such as bulk Hi-C and scHi-C, to map these 3D DNA structures at bulk and single-cell levels, but the resulting data are often incomplete and noisy, making analysis challenging. To address this problem, we developed HiCENT, an advanced artificial intelligence-based tool designed to enhance the quality of 3D DNA maps in both bulk Hi-C and scHi-C datasets. Using deep learning techniques, HiCENT improves resolution, providing researchers with more detailed and accurate genomic data. HiCENT significantly outperformed existing methods, revealing fine-scale DNA structures and improving the accuracy of cell-type identification. By making complex genomic data more accessible and reliable, HiCENT will help scientists uncover new insights into gene regulation, disease mechanisms and DNA organization, contributing to research in cancer, aging and other biological fields where understanding DNA structure is crucial.

## 1. Introduction

Understanding the three-dimensional (3D) organization of the genome in cells is crucial for explaining important chromosomal activities. High-throughput chromosome conformation capture (Hi-C) and its derived technologies have been developed to delineate genome-wide chromatin interactions at population level or in individual cells [1]. Hi-C approaches capture chromatin contacts and measure the contact strengths among chromosomal loci within the genome that are named as contact matrices, providing insights into the 3D organization of the genome [2]. Newly developed single-cell Hi-C (scHi-C) technologies can be used to detect cell-to-cell variations in genome-wide chromatin interactions and have the potential to interrogate chromosome structural heterogeneity in different cell types and states [3,4]. High-resolution Hi-C and scHi-C datasets have helped to reveal chromosomal organization structures such as topologically associating domains (TADs) [5], loop structures [6] and A/B compartments [7], thus contributing to more in-depth studies of genome function.

Current raw contact matrices of chromatin interaction possess relatively low resolution for both bulk and scHi-C data, which are mainly affected by different protocol complexities, the number of cells and sequencing depth [8,9]. For example, in scHi-C experiments, the uniquely captured paired-end reads for each cell are widely observed to be a small proportion of theoretical estimations of chromatin interactions, resulting in very sparse patterns of interacting maps. Certain contacts are structural zeros due to loci truly not interacting, while some contact zeros could be sampling zeros (“dropouts”) due to insufficient sequencing depth and experimental limitations. As a result, many Hi-C and scHi-C experiments have not reached a saturation state to enable high-resolution characterizations of spatial structures and chromatin interactions [10]. This limitation restricts accurate understanding of genomic interactions and may obscure crucial gene regulatory patterns and structural changes [8,11].

Many computational methods have been designed to improve the contact resolution of Hi-C and scHi-C data. Notably, deep learning techniques such as Convolutional Neural Networks (CNNs), autoencoders and Generative Adversarial Networks (GANs) have been used and achieved reasonable precision. For example, HiCPlus [12], HiCNN [13], ReHiC [14] and SRHiC [15] are supervised learning methods based on CNN architecture used to train the mapping between low-resolution and high-resolution Hi-C matrices by refining network architecture and/or increasing network depth. VEHiCLE utilizes a variational autoencoder with an adversarial training strategy and a chromosome topology-inspired insulation loss to enhance contact matrices [16]. DeepLoop utilizes a U-net structure model for data enhancement, accompanied by a 5-layer autoencoder for data denoising [17]. HiCSR [18], DeepHiC [19], hicGAN [20], EnHiC [21] and HiCARN [22] are all GAN-based Hi-C enhancement methods, primarily consisting of generators and discriminators and hybridized with different loss functions. However, due to higher sparsity and noise of scHi-C matrices compared to bulk Hi-C, the enhancement of scHi-C data is more challenging. HiCImpute considers the spatial dependencies of scHi-C 2D data structure while also borrowing information from bulk data and similar single cells [23]. scHiCluster [24], HiC-SGL [25], Higashi [26] and scDEC-Hi-C [27] use imputation methods to enhance data, with the aim of cell clustering. They basically use information from neighbor bins or model reconstruction for data imputation. For example, scHiCluster uses convolution of neighbor bins and long-range random walk to impute scHi-C matrices [24]. HiC-SGL constructs a k-hop neighborhood subgraph for each bin pair for imputation. In scDEC-Hi-C, the reconstructed Hi-C map from the decoder of its autoencoder is regarded as the imputed scHi-C map. Furthermore, several methods increase the number of layers in their models to improve the scHi-C imputation effect. For example, compared to HiCARN, ScHiCEDRN only increases the number of residual blocks in the generator to 32 [28]. In scVI-3D [29], a zero-inflated negative binomial distribution (ZINB) is added to the deep generative model to encode whether a particular locus pair has “dropout” due to technical artifacts.

Although these methods provide diverse solutions for enhancing Hi-C or scHi-C data resolution, there is still significant room for performance improvement in exploring finer genomic structures for Hi-C and ultra-sparse scHi-C data [9]. In recent years, generative transformer models have gained prominence in image generation and enhancement tasks by excelling in the reconstruction of high-resolution details from low-resolution data [30]. Here, we proposed a generative deep learning architecture, HiCENT, for enhancing the resolution of Hi-C and scHi-C data. HiCENT incorporates a CNN backbone to extract fine-grained local spatial features and a transformer backbone to model the complex spatial interaction characteristics of 3D chromatin interaction data. The hybrid architecture of HiCENT allows for the deep extraction of subtle interaction features, capturing long-distance genomic dependencies and reconstructing high-resolution genomic contact maps accordingly. We tested HiCENT on bulk Hi-C and scHi-C data and the results demonstrate its superior enhancement effects compared to five popular methods. In real application, HiCENT significantly enhanced the Hi-C data from the GM12878 cell line to present finer 3D structural features at the scales of TADs and chromosomal loops. It also significantly improved clustering performance for scHi-C datasets of five human cell lines. The results highlight that HiCENT is specifically optimized to capture the intricate features of chromatin organization, enabling it to reconstruct high-quality genomic contact maps with exceptional accuracy and effectiveness.

## 2. Materials and Methods

### 2.1. Overview of HiCENT

**Figure 1:**
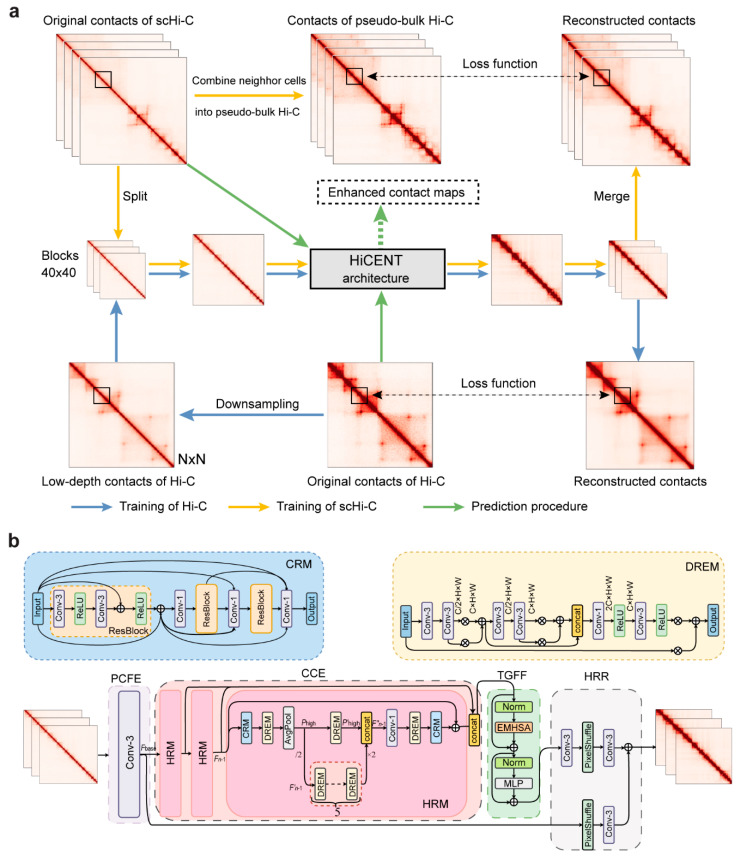
Overview of the HiCENT method. (**a**) Flowchart of the HiCENT pipeline, illustrating the process of enhancing contact maps from low-depth scHi-C and Hi-C data. The workflow includes down-sampling, pseudo-bulk Hi-C generation and reconstruction steps with loss functions applied at multiple stages. (**b**) Detailed deep learning architecture of HiCENT, depicting the different components involved in training and prediction.

HiCENT is a pipeline designed for Hi-C and scHi-C data enhancement, which includes different data preprocessing programs and deep generative transformer models. The flowchart of the HiCENT pipeline is shown in Figure 1a. For Hi-C data, HiCENT adopts a random down-sampling strategy to generate low-resolution (or low-depth, LD) contact maps as training datasets from original depth (OD) contact maps. For scHi-C data, HiCENT fully addresses its ultra-sparsity by combining similar cells into pseudo-bulk Hi-C sets as OD contact maps. Given the large size of a genome-scale contact map, HiCENT splits it into blocks of 40×40 pixels for training and testing and then merges the enhanced blocks into a whole contact map. Once the HiCENT model has been trained, the original contact maps can be spilt and input into the model for enhancement.

The deep learning architecture of HiCENT is mainly composed of four core modules (Figure 1b): an initial convolutional feature extraction module that extracts basic features from low-resolution data, a convolutional context enhancement module that enhances these features via advanced residual modules, a global feature fusion module that utilizes the powerful capabilities of transformers to integrate and optimize the enhanced features and a high-resolution reconstruction module that ultimately generates refined high-resolution Hi-C data. The integration of these modules in HiCENT not only improves data quality, but also significantly deepens the interpretive power of the model, revealing the complex structural dynamics within the genome.

### 2.2. HiCENT Architecture

HiCENT combines the advantages of CNN and transformer modules to enhance Hi-C or scHi-C data by utilizing their respective strengths in feature extraction and capturing long-distance dependencies. The hybrid architecture of HiCENT is designed to effectively extract core features from input Hi-C data while maintaining sufficient network depth to achieve large model capacity. HiCENT mainly consists of four modules, some of which are composed of multiple sub-modules, as shown in Figure 1b.

The first module is called “Preliminary Convolutional Feature Extraction Module” (PCFE), which aims to extract potential base feature maps $F_{base}$ from the input low-depth Hi-C or scHi-C data, $I_{LD}$, preparing for subsequent feature processing. PCFE only contains a 3×3 convolutional layer $Conv_{3\times 3}$, that is, $F_{base}=Conv_{3\times 3}(I_{LD})$.

The second module is called the “Convolutional Context Enhancement Module” (CCE), which is used to enhance the base features $F_{base}$ extracted by the PCFE module to local contextual features $F_{local}$. The CCE module is composed of several sub-modules known as Hyper Residual Modules (HRMs) that are connected sequentially to extract and refine hierarchical contextual information. The transformation of base features through the HRMs can be expressed as:

(1) $$F_{n}=\xi^{n}\left(\xi^{n-1}\left(\ldots \xi^{1}\left(F_{base}\right)\right)\right),$$

where $\xi^{n}$ denotes the mapping of n-th HRM and $F_{n}$ the output of n-th HRM. In practice, we utilized 3 HRMs. All outputs of these HRMs are concatenated to $F_{local}=[F_{1},F_{2},\ldots ,F_{n}]$, which represents the enhanced local contextual features and will be sent to the third module.

The third module is called “Transformer Global Feature Fusion Module” (TGFF), which utilizes transformer structures, including an Efficient Multi-Head Self-Attention (EMHSA) and a Multi-Layer Perceptron (MLP), to capture long-distance dependencies between features and output $F_{global}$.

(2) $$F_{global}=\psi \left(F_{local}\right),$$

where $F_{global}$ is the output of the TGFF module, and ψ represents the operations of the module.

Finally, $F_{global}$ and $F_{\mathrm{base}}$ are simultaneously fed into the last module to reconstruct a high-resolution (HR) contact map $I_{HR}$. The last module is called the “High-Resolution Reconstruction Module” (HRR), which can be written as:

(3) $$I_{HR}=\varphi \left(\varphi_{p}\left(\varphi \left(F_{global}\right)\right)\right)+\varphi \left(\varphi_{p}\left(F_{base}\right)\right),$$

where φ and $\varphi_{p}$ represent the 3×3 convolution layer and the Pixel-Shuffle layer [31], respectively.

In the second module (CCE), as shown in Figure 1b, each HRM mainly consists of Cascading Residual Modules (CRMs) and Dynamic Residual Enhancement Modules (DREMs). At the beginning of an HRM, a CRM is used to process input features $F_{n-1}$ and establish residual connections to capture complex feature patterns within the input Hi-C data. Next, a DREM is used to facilitate feature extraction and transformation, supporting high-resolution representation of input Hi-C data. Subsequently, $F_{n-1}$ is down-sampled to $F_{n-1}^{\prime }$ and an average pooling layer is used to capture high-frequency features $P_{high}$ within the input contact map, aiming to extract and highlight both local and global interaction patterns within the genome. Afterwards, a single DREM is used to process the $P_{high}$ to align the feature space with $F_{n-1}^{\prime }$ and output $P_{high}^{\prime }$. Meanwhile, five DREMs are reused on $F_{n-1}^{\prime }$ to progressively extract features and a reconstructed $F_{n-1}^{\prime }$ is up-sampled to the original size of $F_{n-1}$ by bilinear interpolation. It is worth noting that the five DREMs share weights to reduce parameters. After that, $F_{n-1}^{\prime }$ and $P_{high}^{\prime }$ are concatenated to obtain features $F_{n-1}^{\prime \prime }$ with preservation of the initial details. Finally, a 1×1 convolution layer, a DREM and a CRM are successively used to reduce the channel number and extract the final features $F_{n}$. Furthermore, the input original feature $F_{n-1}$ is added to $F_{n}$ for stabilizing the training.

Each CRM mainly consists of three Residual Network Blocks (ResBlocks). Each ResBlock contains two 3×3 convolutional layers, each followed by the ReLU activation. The output of each ResBlock is cascaded with its corresponding input tensor and linearly transformed through a 1×1 convolutional layer to reduce feature dimensions and control model complexity. This cascading ResBlock structure aids in capturing and refining multi-scale and multi-level feature information, thereby producing more expressive feature representations and providing enhanced performance for the model. Each DREM contains two residual units, a 1×1 convolutional layer and a 3×3 convolutional layer. Each residual unit consists of two 3×3 convolutional layers associated with a residual scaling with two adaptive weights, used to adjust the importance of the residual path and identity path. The outputs of two residual units are concatenated, followed by two convolutional layers, which are used to extract hierarchical and valid information from the fused features.

The TGFF module, which leverages the robust capability of the transformer model for feature expression and long-range dependency modeling, is used to recover lost details and decrease blurriness between long-distance genomic regions in low-depth Hi-C data. The TGFF module only employs the encoder structure of the standard transformer, and mainly consists of two blocks, an MHSA and an MLP. Layer-normalization is called before running each of the two blocks and the residual connection is used after each block is run. Formula (2) can be rewritten as:

(4) $$F_{m}=EMHSA\left(Norm\left(F_{\mathrm{local}}\right)\right)+F_{local}{,\;F}_{global}=MLP\left(Norm\left(F_{m}\right)\right)+F_{m}$$

where EMHSA(⋅) and $MLP\left(\cdot \right)$ represent the EMHSA and MLP operations. The MLP consists of two linear layers interspersed with the activation function “SwiGLU” [32].

The EMHSA is modified from the classic Multi-Head Attention (MHA) to adapt highly sparse Hi-C data. EMHSA focuses on significant interactions within sparse Hi-C data, namely those with high attention scores, to facilitate the identification and restoration of these interactions and balance the capture of long-distance interactions with attention to local regions. First, the number of channels of the feature map $F_{local}$ is halved via a reduction layer, and then the reduced feature map $F_{local}^{\prime }$ is projected via a linear layer onto three elements: Q (Query), K (Keys) and V (Values). Suppose the shape of $F_{local}^{\prime }$ is B×N×C, where B is the batch size, N is the sequence length (i.e., the size of the input square contact matrix) and C is the number of channels. After performing linear projection and MHA with m heads, we reshaped Q, K and V and permuted the shape to $B\times m\times N\times \frac{C}{m}$. To reduce the computational time and GPU memory cost required for calculating the self-attention matrix with shape B×m×N×N, a Feature Split Module is used to split Q, K and V each into s equal segments with shape $B\times m\times \frac{N}{s}\times \frac{N}{s}$, denoted as $Q_{1},\ldots ,Q_{s}$, $K_{1},\ldots ,K_{s}$, and $V_{1},\ldots ,V_{s}$. For each triplet $\left(Q_{i},K_{i},V_{i}\right),$ a Scaled Dot-Product Attention (SDPA) operation defined as

(5) $$\mathrm{Attention}(Q,K,V)=\mathrm{softmax}\left(\frac{Q_{i}K_{i}^{T}}{\sqrt{d_{k}}}\right)V_{i}$$

is calculated. All outputs of the s SDPA operations are concatenated together to obtain the whole output features (s=5, in our practice). At the end of the EMHSA, an Expansion layer is used to recover the number of channels.

### 2.3. Loss Functions in HiCENT

To effectively enhance the resolution of Hi-C and scHi-C data, a comprehensive loss function is used to train the model, ensuring that the generated contact matrix is visually and biologically aligned with the original target contact matrix. First, to enhance sensitivity to details and robustness to outliers, $l_{1}$ loss function $L_{l_{1}}$ and mean squared error (MSE) loss function $L_{MSE}$ are combined as the reconstruction loss of the model. $L_{l_{1}}$ function computes the average absolute difference and $L_{MSE}$ function computes the average squared difference between the reconstructed HR contact elements (pixels)$\;{\left\{R_{i}\right\}}_{i=1}^{N}$ and OD contact elements (pixels) ${\left\{O_{i}\right\}}_{i=1}^{N}$, as shown in Equations (6) and (7).

(6) $$L_{l_{1}}=\frac{1}{N}\sum_{i=1}^{N}\left|O_{i}-R_{i}\right|$$

(7) $$L_{MSE}=\frac{1}{N}\sum_{i=1}^{N}{\left(O_{i}-R_{i}\right)}^{2}$$

Second, a Feature Consistency (FC) Loss $L_{FC}$ is defined by computing the MSE between the high-level features extracted from the reconstructed matrix R and OD matrix O using a pretrained ResNet model:

(8) $$L_{\mathrm{FC}}=\mathrm{MSE}(F(O),F(R))$$

where *F*(⋅) denotes the feature extraction function of the ResNet50 model. The FC loss function is expected to enhance the capability for extracting intrinsic biological features.

Third, to suppress noise and enhance the coherence of interactions in the reconstructed contact matrix, a Total Variation (TV) Loss function $L_{TV}$ is defined as:

(9) $$L_{\mathrm{TV}}=\frac{2\phi }{B}\left(L_{VV}+L_{HV}\right),$$

where B is the batch size, ϕ is a weight scalar, $L_{VV}$ represents the Vertical Variation (VV) Loss defined in Equation (10) and $L_{HV}$ represents the Horizontal Variation (HV). Loss defined in Equation (11), if the reconstructed square contact matrix is ${\left(R_{i,j}\right)}_{H\times H}.$

(10) $$L_{VV}=\frac{1}{\left(H-1\right)H}\sum_{i=1}^{H-1}\sum_{j=1}^{H}{\left(R_{i+1,j}-R_{i,j}\right)}^{2}$$

(11) $$L_{VV}=\frac{1}{H(H-1)}\sum_{i=1}^{H}\sum_{j=1}^{H-1}{\left(R_{i,j+1}-R_{i,j}\right)}^{2}$$

Ultimately, the total loss function $L_{total}$ of the HiCENT model is defined as the weighted sum of the aforementioned loss functions:

(12) $$L_{total}=L_{MSE}+L_{l_{1}}+\lambda_{1}L_{\mathrm{FC}}+\lambda_{2}L_{\mathrm{TV}},$$

where $\lambda_{1}$ and $\lambda_{2}$ are adjustable parameters that can be tuned based on the training performance.

### 2.4. Hi-C and scHi-C Datasets Used for Enhancement

We used Hi-C data created by Rao et al. [6], which were generated from the human lymphoblastoid cell line GM12878, the erythroleukemia cell line K562 and the mouse B-cell lymphoma cell line CH12-LX. The data were downloaded from the Gene Expression Omnibus (GEO) database with accession number GSE63525. The data were processed and used following the same procedures used in HiCARN [22]. We tested HiCENT on scHi-C datasets from five human cell lines: embryonic stem cells (hESC) H1, HFF-hTERT clone #6 (HFFc6), GM12878, IMR90 and HAP1 cells. The datasets were obtained through the 4D Nucleome Consortium (https://www.4dnucleome.org/cell-lines/, 1 June 2024) and parsed into nine different single-cell combinatorial indexed Hi-C (sci-Hi-C) libraries, consisting of over 19,000 cells. The contact matrices of each cell, using bins of 500 kb, were directly downloaded from https://noble.gs.washington.edu/proj/schic-topic-model/ (1 June 2024) [4]. We named the dataset “4DN sci-Hi-C” in this research.

### 2.5. Data Preprocessing

For the preprocessing of Hi-C data, HiCENT follows the same steps as DeepHiC [19]. In brief, we first filtered out low-quality data with a Mapping Quality (MAPQ) score less than 30 to ensure high data quality, and then employed the KR normalization method [33] to standardize the Hi-C contact matrices, aiming to eliminate potential biases and outliers and enhance the consistency and reliability of the data. We then performed data format conversion to ensure that the Hi-C contact matrices were transformed into a format suitable for deep learning model processing. To generate the low-depth datasets required for training, we adopted a random down-sampling strategy. Low-depth data were simulated by randomly down-sampling the sequencing reads by different ratios of 1/16, 1/32, 1/64 and 1/100. This down-sampling strategy simulates the low-resolution data scenarios encountered in real Hi-C experiments, providing a basis for evaluating the performance and robustness of the model. The contact maps of low-depth data were processed at lower resolution (i.e., larger bin size). Low-resolution contact maps were constructed here using the same bin size as the OD contact matrix.

For preprocessing of scHi-C data, we used mapped reads as provided by the authors [4]. The processed read pairs were mapped to the human genome assembly hg19 using Bowtie2 with default settings [34], and aligned reads were filtered out with MAPQ <30. Cells with <1000 unique reads, an intra/inter-chromosomal contact ratio lower than 1, or <95% of uniquely mapped reads, were also filtered out. Due to the extreme sparsity of scHi-C contact maps, we only considered enhancing contacts with bins of 500kb. Furthermore, to obtain a contact matrix with higher depth for each cell, HiCENT selects the contact matrices of the top k cells of the same cell type (i.e., highest correlation scores) and stacks them together to form a pseudo-bulk Hi-C matrix.

For datasets from cell lines, we excluded the sex chromosomes and separated the 22 autosomes into three groups, 14 for the training set, 4 for the validation set and the remaining 4 for the testing set. To assess the performance of our HiCENT model and evaluate its generalizability across different chromosomes, cell types and species, we tested the model on the testing set of 4 chromosomes from the same cell line as well as data from the other cell lines.

### 2.6. Implementations of HiCENT and Six Other Methods

HiCENT was trained on 40×40 sub-matrices in 20 epochs using Adam optimizer with a batch size of 16 and an initial learning rate of $2\times 10^{-4}.$ The subsequent learning rate was reduced to half of its previous value every 5 epochs. The default number of channels is 32 and the number of heads m in EMHSA is set to 8. The other settings of the HiCENT model are shown in Figure 1b. To benchmark the performance of HiCENT, we selected five Hi-C enhancement methods (HiCPlus, DeepHiC, HiCNN, HiCSR and HiCARN) known for their effectiveness and architectural diversity. Additionally, we included ScHiCEDRN, which is specifically designed for scHi-C data enhancement. This comprehensive selection ensures a robust comparative analysis across both Hi-C and scHi-C datasets (detailed information on these tools is listed in Table S1). HiCPlus and HiCNN were run with their pre-trained model parameter files separately, which were packaged together with the source code provided by their authors. All tools were tested according to the parameter settings in their corresponding papers performed on a Linux workstation (CPU: Intel Xeon E5-2620/2.10GHz/8cores) with an NVIDIA RTX4090 GPU with 24 Gb of memory. The detailed components and parameters of all seven models are listed in Table S2.

### 2.7. Performance Evaluation Metrics

Several image evaluation metrics, including MSE, the Structural Similarity (SSIM) index and Peak Signal to Noise Ratio (PSNR), were employed to evaluate the HiCENT model and the five other methods. These metrics have been used in other Hi-C enhancement tools such as HiCSR and HiCARN. If x and y represent the real HR target and the enhanced contact map, the formulas for computing SSIM and PSNR are shown in Equations (13) and (14), where $\mu_{x}$ and $\mu_{y}$ are the means, $\sigma_{x}^{2}$ and $\sigma_{y}^{2}$ are the variances, and $\sigma_{xy}$ is the covariance of x and y. $C_{1}$ and $C_{2}$ are two constants with default values ${\left(0.01\right)}^{2}$ and ${\left(0.03\right)}^{2}$.

(13) $$SSIM\left(x,\;y\right)=\frac{\left(2\mu_{x}\mu_{y}+C_{1}\right)\left(2\sigma_{xy}+C_{2}\right)}{\left(\mu_{x}^{2}+\mu_{y}^{2}+C_{1}\right)\left(\sigma_{x}^{2}+\sigma_{y}^{2}+C_{2}\right)},$$

(14) $$PSNR=10\times {log}_{10}\left(\frac{N}{\sqrt{MSE}}\right),$$

SSIM is used to measure the similarities between two contact maps and PSNR is used to measure the degree of noise removal. The higher the PSNR value, the more noise is removed. The implementation of SSIM in DeepHiC and the implementations of MSE and PSNR in HiSCR were used here and calculated for each 40×40 sub-matrix predicted by HiCENT and the five reference methods.

Since the metrics described above are mainly designed for evaluating the perceptual quality of natural images, they cannot well account for the unique spatial structure inherent in Hi-C data [18]. Therefore, we also employed two Hi-C specific metrics, GenomeDISCO [35] and HiCRep [36], which quantify the reproducibility of Hi-C samples with different perspectives. First, GenomeDISCO performs random walks on a network created from the Hi-C data to smooth the contact maps and then computes the similarity scores between the smoothed maps. Second, HiCRep performs a 2D mean filter to smooth each contact map, stratify each smoothed map by genomic distance and then compute the strata-weighted correlation between contact maps. The SSIM, GenomeDISCO and HiCRep scores all range from 0 to 1, where larger values indicate higher similarity between two contact maps.

We also tested HiCENT to see how its enhanced scHi-C data might help to improve cell clustering. For the enhanced data, we performed the PCA analysis that is implemented in the SCANPY pipeline and selected the top 40 most significant principal components. We then used the Leiden algorithm with default parameters in SCANPY for clustering. The clustering performance was compared among six methods: HiCENT, scHiCluster, Higashi, scDEC-Hi-C, HiC-SGL and ScHiCEDRN. The clustering effect of these methods was evaluated by the adjusted Rand index (ARI).

## 3. Results

### 3.1. Hyperparameter Selections of HiCENT

**Figure 2:**
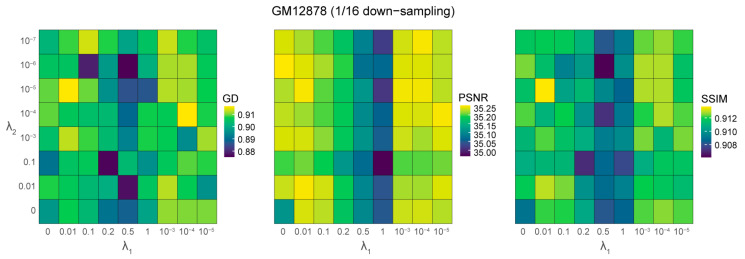
Performance evaluation of HiCENT under different values of the hyperparameter pair $\lambda_{1}$ and $\lambda_{2}$ for GM12878 at 1/16 down-sampling. The heatmaps display the scores for GenomeDISCO (GD), PSNR and SSIM, where higher values indicate better performance.

HiCENT includes two hyperparameters, $\lambda_{1}$ and $\lambda_{2}$, in its loss function, which influence the model’s ability to reconstruct and enhance Hi-C contact maps. To systematically assess the impact of these hyperparameters on program performance, we conducted a grid search by testing various values of $\lambda_{1}$ and $\lambda_{2}$ on the 1/16 down-sampling of the GM12878 Hi-C dataset (Figure 2). The optimal configuration was identified as $\lambda_{1}=0.01$ and $\lambda_{2}=1e-5$, which yielded the highest GenomeDISCO score (~0.9166) and SSIM score (~0.9137) while maintaining a high PSNR (~35.25). These results indicate that this setting effectively balances the trade-off between reconstruction fidelity and noise suppression in down-sampled Hi-C data. Additionally, overly large or small values of $\lambda_{1}$ and $\lambda_{2}$ lead to suboptimal performance, reinforcing the importance of carefully tuning these hyperparameters for optimal Hi-C data enhancement.

We conducted an extensive analysis of the training process by varying the number of epochs and monitoring key evaluation metrics (Figure S1). The results indicate that all metrics exhibit a rapid improvement in the initial training phase, with noticeable stabilization occurring around 20 epochs. Specifically, the MSE and total loss curves show a sharp decrease in the first 20 epochs, followed by minimal fluctuations, suggesting convergence. Similarly, PSNR and SSIM values reach their peak stability at approximately 20 epochs, with only marginal variations observed beyond this point. Based on these coincident observations, we set 20 epochs as an optimal stopping point for model training.

### 3.2. HiCENT Outperforms Reference Methods in Image-Based Metrics and Hi-C Reproducibility Metrics

We evaluated the HiCENT and five reference methods (HiCPlus, DeepHiC, HiCNN, HiCSR and HiCARN) on Hi-C datasets with different down-sampling resolutions using three image-based metrics: SSIM, PSNR and MSE. Low-depth data were simulated by randomly down-sampling the Hi-C sequencing reads of the human-derived GM12878 cell line using 4 different ratios: 1/16, 1/32, 1/64 and 1/100. The bin size for constructing the contact matrices for both low-depth data and original data are 10 Kb. The experimental results of SSIM, PSNR and MSE indicate that HiCENT has advantages in restoring and enhancing the resolution (depth) of Hi-C data, outperforming the five reference methods confirmed by image evaluation (Table 1). Especially, the PSNR scores are higher than the five reference methods at all down-sampled ratios, indicating that HiCENT reduces more noise during the enhancement process. Furthermore, we found that HiCENT SSIM and PSNR scores remain highest compared to the other methods with minimal attenuation during the decrease in down-sampling ratio from 1/16 to 1/100. These results demonstrate the effectiveness and robustness of the HiCENT model in reconstructing high-fidelity genomic data from extremely low-resolution inputs and suggest that it may also be effective for ultra-low-resolution scHi-C data.

We also compared HiCENT with the reference methods using two Hi-C reproducibility metrics: GenomeDISCO and HiCRep. These metrics are mainly used to measure concordance in spatial structures between two contact matrices. Among 22 autosomes of the GM12878 cell line, 4 chromosomes, Chr4, Chr14, Chr16 and Chr20, are used as the test set. As shown in Table 2 and Table 3, HiCENT achieves higher GenomeDISCO and HiCRep scores than the five reference methods on each of the four tested chromosomes with 1/16 down-sampled ratio. The average scores are also greater, overall showing that HiCENT consistently outperforms the other methods. Importantly, the highest average scores of the two Hi-C reproducibility metrics in HiCENT also indicate that HiCENT has significant advantages in capturing and recovering 3D chromosomal structures.

HiCENT demonstrates remarkably fast training speed and efficient memory usage compared to other competing models (see details in Table S3). On the GM12878 dataset, HiCENT completes training in just 1.96 h, making it the fastest among all tested models. In contrast, HiCNN and HiCSR require significantly longer training times of 24.78 h and 19.93 h, respectively. Additionally, HiCENT maintains a relatively low memory footprint, utilizing only 15.72% of the 24 GB GPU memory, whereas HiCNN consumes the highest memory at 40.67%, and HiCPlus uses the least at 6.24%. HiCENT’s efficient resource allocation enables it to balance speed and memory consumption effectively. These results highlight HiCENT’s advantage in computational efficiency, making it a highly scalable and practical choice for chromatin interaction data enhancement.

**Figure 3:**
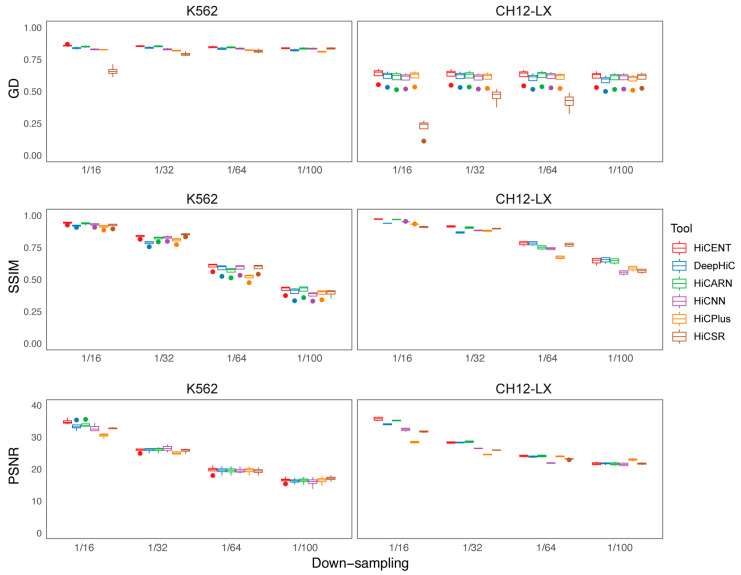
Performance comparison of six methods at different down-sampling levels for K562 and CH12-LX cell lines. GenomeDISCO (GD), SSIM and PSNR scores were calculated to evaluate the performance of HiCENT, DeepHiC, HiCARN, HiCNN, HiCPlus and HiCSR across varying levels of data sparsity. Detailed results are provided in Table S4 for GD scores, Table S5 for SSIM scores and Table S6 for PSNR scores.

In addition, we conducted training, validation and testing experiments on two other cell lines, K562 and CH12-LX, with 1/16, 1/32. 1/64 and 1/100 down-sampled ratios. We calculated the average scores of GenomeDISCO, SSIM and PSNR for a total of six methods and found that HiCENT consistently outperforms all competing methods across multiple evaluation metrics (Figure 3). Specifically, HiCENT achieves the highest GenomeDISCO scores across all down-sampling ratios for both the K562 and CH12-LX cell lines (Table S4). For SSIM, HiCENT achieves the highest scores in nearly all cases, demonstrating its ability to maintain structural similarity and spatial fidelity, particularly for the highest, most challenging down-sampling conditions (Table S5). In PSNR evaluations, HiCENT exhibits the best performance at the 1/16 down-sampling ratio for both K562 (35.0863) and CH12-LX (35.9764), while also maintaining competitive scores across other down-sampling levels (Table S6). Notably, performance remains robust even at extreme down-sampling ratios (1/64 and 1/100), outperforming other methods in GenomeDISCO and SSIM, and further underscoring its effectiveness in handling highly sparse Hi-C data. These findings highlight the ability of HiCENT to enhance low-resolution Hi-C contact maps with superior structural preservation and denoising capabilities and demonstrate it to be a powerful tool for improving data quality in both bulk and single-cell Hi-C applications.

### 3.3. Visual Comparison of Predicted Contact Maps in Hi-C Data

**Figure 4:**
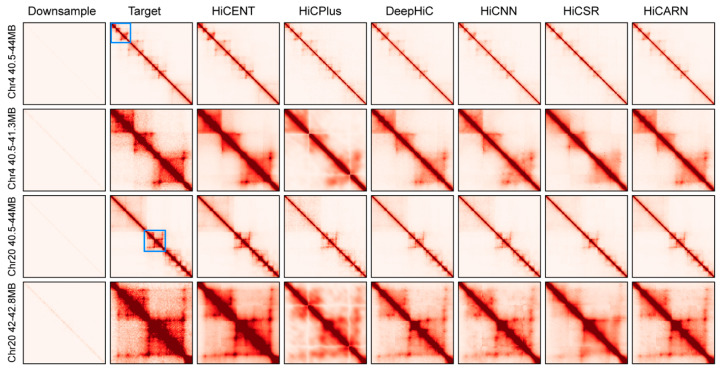
Visual comparison of enhanced contact matrices generated by different methods. Two genomic regions from the GM12878 cell line are displayed along with their zoomed-in subregions (highlighted by blue rectangles) to illustrate fine-scale structural differences.

As an example of the usefulness of HiCENT for reconstructing 3D organizations, we performed a visual comparison of reconstructed contact maps for Chr4 and Chr20 from the test set of the GM12878 cell line. Figure 4 shows the heatmaps for the 40.5–44 MB region of Chr4 and the 40.5–44 Mb region of Chr20 at down-sampled ratio 1/16. Results indicate that the contact maps predicted by HiCENT have the least difference from the corresponding target maps in both chromosomal regions. Unlike other methods that introduce artifacts or over-smooth Hi-C contacts, HiCENT retains key interaction domains and structural patterns with minimal loss of resolution.

Zooming in separately on the 40.5–41.3 Mb sub-region of Chr4 and the 42–42.8 Mb sub-region of Chr20, the detailed patterns of intra-interactions output by HiCENT are more clearly visible compared with the reference methods, which were mostly blurred or missing in the down-sampled images. In the sub-regions of the target contact maps, high-contrast square regions which are symmetrical along the diagonal are often TADs. Moreover, if the angles of these squares are highlighted, they are likely to be loops. The result, at both large and fine resolution, highlights the superior performance of HiCENT in reconstructing high-resolution chromatin interaction maps from low-coverage data and demonstrates its robustness in enhancing sparse Hi-C datasets while minimizing noise and preserving structural patterns.

### 3.4. HiCENT Enhanced scHi-C Data Facilitates Cell Clustering

**Figure 5:**
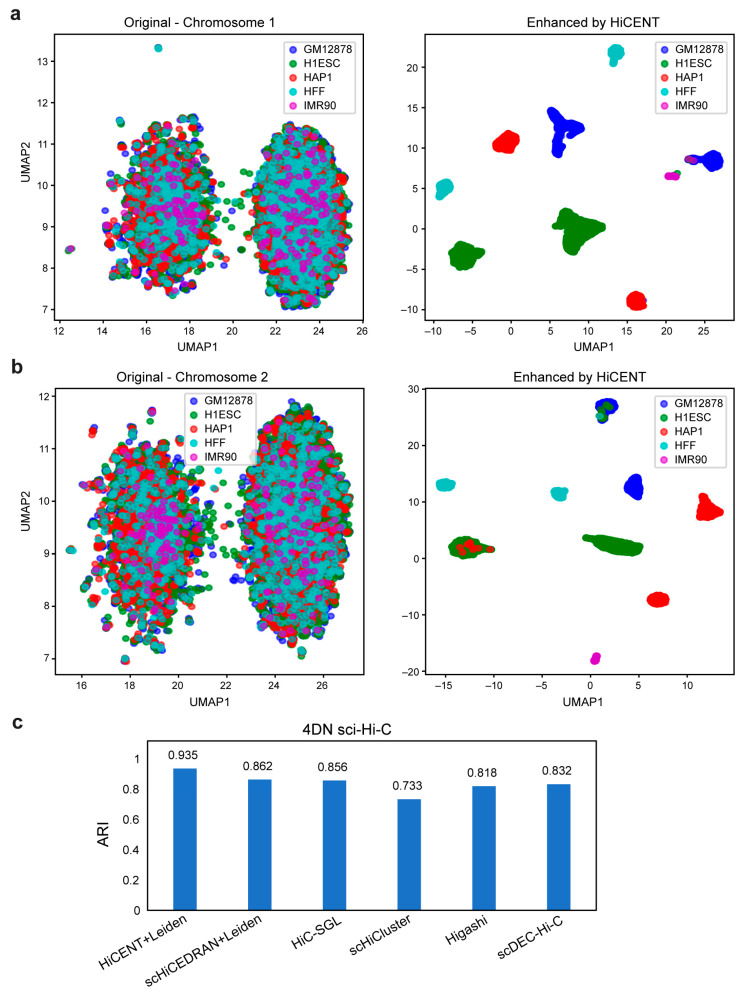
HiCENT-enhanced scHi-C data improves clustering analysis. (**a**,**b**) UMAP plots of original scHi-C data (left) and HiCENT-enhanced data for Chr1 and Chr2 of the 4DN sci-Hi-C dataset, respectively. (**c**) ARI scores for comparing different clustering methods on the 4DN sci-Hi-C dataset. The results for scHiCluster, Higashi, scDEC-Hi-C and HiC-SGL were directly referenced from the HiC-SGL study [25].

For scHi-C data, the high sparsity of the contact matrix for each cell means that down-sampling could result in complete loss of important biological signals. To enhance scHi-C matrices, we first calculated the Pearson correlation coefficient between each pair of cells and identified the top k=10 nearest neighbors to construct a pseudo-bulk Hi-C matrix for each cell as its original-depth map. This can be regarded as a down-sampled map. We performed HiCENT on scHi-C data from five human cell lines (GM12878, H1Esc, HFF, IMR90 and HAP1). Unlike the pipeline for bulk Hi-C data analysis, we divided all cells into three equally sized sets for training, validation and testing. After the model was trained, we performed UMAP dimensionality reduction on the chromosomes from the cells in the test set (Figure 5). We found that the enhanced data allowed high discrimination among different cell types, whereas it was difficult to distinguish different cell types on the UMAP plots of the original data. UMAP plots of original Chr1 and Chr2 scHi-C data in Figure 5a,b show that different cell types are mixed together, with evident batch effects due to data coming from different libraries.

To test if HiCENT enhancement can improve downstream computational analysis, we used the Leiden clustering method on the enhanced data. We compared its performance with other popular clustering methods, including scHiCluster, Higashi, scDEC-Hi-C and HiC-SGL as they have been recently used for performance validation on the 4DN sci-Hi-C datasets [25]. Since HiCENT and ScHiCEDRN were not designed for scHi-C clustering, we first trained HiCENT and ScHiCEDRN on the 4DN sci-Hi-C dataset using three-fold cross validation to obtain the final enhanced maps for all cells. Then, we used the PCA in the SCANPY pipeline to select the top 40 principal components and then used the Leiden algorithm with default parameters in SCANPY for clustering. We found ARI scores of 0.935 for HiCENT and 0.862 for ScHiCEDRN (Figure 5c). These results confirm that HiCENT-enhanced scHi-C data facilitates cell clustering.

## 4. Discussion

In this study, HiCENT represents a significant advancement in the enhancement of chromatin interaction data, particularly for low-resolution Hi-C and sparse scHi-C datasets. HiCENT introduces a novel hybrid architecture combining a CNN backbone for fine-grained spatial feature extraction with a transformer backbone for modeling complex long-distance genomic interactions. This design enables HiCENT to effectively capture subtle interaction features and intricate chromatin structures, achieving superior resolution enhancement for both bulk Hi-C and sparse scHi-C data. Its outstanding performance across multiple metrics, such as SSIM, PSNR, MSE, GenomeDISCO and HiCRep, underscores its ability to reconstruct high-resolution contact maps more effectively than five widely used reference methods. Real-world applications, including bulk Hi-C and scHi-C data from the GM12878, K562, CH12-LX, H1Esc, HFF, IMR90 and HAP1 cell lines, demonstrate that HiCENT has superior ability to recover 3D chromosomal structures, enabling improved biological applications, such as for cell clustering. These results highlight the performance of HiCENT to enhance both bulk and single-cell Hi-C data resolution and quality, facilitating diverse downstream studies of 3D chromatin organization and associated functions.

Despite remarkable performance in enhancing chromatin interaction data, there remain areas for further optimization and broader application of HiCENT. Future studies could explore integrating adaptive learning strategies or meta-learning approaches [37,38,39] to improve generalizability across diverse datasets and experimental conditions. It is also valuable to validate the performance of HiCENT across a range of bin sizes and sequencing depths. This investigation will provide valuable insights into the optimal bin size and sequencing depth required for accurate chromatin interaction enhancement, further benchmarking HiCENT’s robustness and generalizability across various Hi-C and scHi-C datasets, thereby facilitating its application in broader genomic studies. Additionally, optimizing HiCENT for ultra-sparse single-cell multi-omics datasets could unlock deeper biological insights by integrating diverse modalities like transcriptomics and epigenomics [40,41]. While our data show that HiCENT significantly enhances cell clustering, its potential applications to other downstream analyses, such as batch correction and integration [42], inferring chromatin loops and regulatory network reconstruction [26,43,44], remains an avenue for future exploration. For example, batch effects across same cell types are clearly observed in the UMAP plots of scHi-C data (Figure 5a,b). Developing efficient methodologies to mitigate these batch effects before clustering would be valuable. Deep learning approaches, such as autoencoders used in scRNA-seq analysis [42], could be similarly adapted to correct scHi-C signals. In this way, developing task-specific extensions of HiCENT for these analyses could open new avenues for investigating 3D genome organization and its functional relevance in biology.

## 5. Conclusions

This study introduces HiCENT, a novel computational method that utilizes advanced deep learning strategies. HiCENT demonstrates high computational efficiency and outperforms existing methods. When applied to real Hi-C datasets, it successfully recovered fine-scale topologically associated domains and chromosomal loops, while it significantly improved clustering performance across different cell lines in scHi-C data. HiCENT software (version 1.0) is well-designed with a user-friendly interface, allowing it to accept interaction matrices as input and generate enhanced matrices as output. Thus, HiCENT represents an essential step in data imputation and will benefit diverse downstream computational tasks, including cell-type identification, regulatory network reconstruction and chromatin loop inference. As scHi-C experiments are inherently complex and challenging, this method facilitates their broader application by enhancing sparse datasets. With the increasing availability of Hi-C and scHi-C data, we believe that HiCENT’s widespread adoption will not only contribute to the development of computational tools for various research topics but also enable novel and more accurate biological discoveries.

## Figures and Tables

**Table 1:** Performance comparison of different Hi-C enhancement methods on the GM12878 cell line at various down-sampled ratios. SSIM, PSNR and MSE scores are shown for six methods evaluated on Hi-C data from the GM12878 cell line across four down-sampled ratios. All scores are the average scores predicted for all sub-matrices in all chromosomes. The optimal score for each criterion among the six methods is highlighted in bold.

	1/16 Down-Sampled			1/32 Down-Sampled		
Model	SSIM	PSNR	MSE	SSIM	PSNR	MSE
HiCPlus	0.8763	31.1084	0.0008	0.8759	32.2933	0.0006
DeepHiC	0.8979	34.5182	0.0003	0.8838	34.0568	0.0004
HiCNN	0.8997	33.8231	0.0004	0.8831	32.6867	0.0006
HiCSR	0.9016	30.8811	0.0009	0.8782	33.1212	0.0005
HiCARN	0.9097	35.1358	0.0003	0.8969	34.3054	0.0003
HiCENT	**0.9152**	**35.2673**	**0.0003**	**0.9026**	**34.4197**	**0.0003**
	1/64 down-sampled			1/100 down-sampled		
Model	SSIM	PSNR	MSE	SSIM	PSNR	MSE
HiCPlus	0.8491	30.94	0.0008	0.8436	30.8603	0.0008
DeepHiC	0.8709	32.6925	0.0005	0.8528	32.0369	0.0007
HiCNN	0.8699	32.1255	0.0007	0.8609	32.0144	0.0006
HiCSR	0.8676	32.3657	0.0006	0.8616	32.0048	0.0006
HiCARN	0.8843	33.4657	0.0005	0.8756	32.9561	0.0005
HiCENT	**0.8899**	**33.5145**	**0.0005**	**0.8828**	**33.0139**	**0.0005**

**Table 2:** GenomeDISCO scores for the GM12878 cell line at a 1/16 down-sampled ratio. The optimal score for each row among the six methods is highlighted in bold.

Model	HiCPlus	DeepHiC	HiCNN	HiCSR	HiCARN	HiCENT
Chr4	0.8782	0.9013	0.8928	0.8832	0.9122	**0.9162**
Chr14	0.8869	0.9102	0.9051	0.897	0.9195	**0.923**
Chr16	0.865	0.89	0.882	0.7311	0.9027	**0.9062**
Chr20	0.8907	0.9157	0.9117	0.9043	0.9235	**0.9275**
Average	0.8802	0.9043	0.8979	0.8539	0.914475	**0.9182**

**Table 3:** HiCRep scores for the GM12878 cell line at a 1/16 down-sampled ratio. The optimal score for each row among the six methods is highlighted in bold.

Model	HiCPlus	DeepHiC	HiCNN	HiCSR	HiCARN	HiCENT
Chr4	0.7953	0.8523	0.8337	0.8716	0.8666	**0.8754**
Chr14	0.8857	0.9158	0.9098	0.9317	0.9278	**0.9340**
Chr16	0.8813	0.9144	0.8989	0.9135	0.9223	**0.9262**
Chr20	0.8776	0.9158	0.9076	0.9255	0.9213	**0.9124**
Average	0.8600	0.8995	0.8875	0.9106	0.9095	**0.9120**

## Data Availability

Datasets used in this study are available in NCBI GEO database with the accession number GES63525 and scHi-C datasets at https://noble.gs.washington.edu/proj/schic-topic-model/ (accessed on 1 June 2024). The programming code for HiCENT and its implementation instructions are available at https://github.com/shaoqiangzhang/HiCENT (accessed on 1 March 2025).

## Supplementary Materials

The following supporting information can be downloaded online: https://www.mdpi.com/article/10.3390/biology14030288/s1. Supplementary File S1 includes Tables S1–S6 and Figure S1. Table S1. Summary of Hi-C and scHi-C enhancement methods evaluated in this study; Table S2. The components and parameters of all models; Table S3. Training times and memory usage of each competing model on GM12878; Table S4. Average scores of GenomeDISCO on the test sets of the K562 and CH12-LX cell lines with different down-sampled ratios; Table S5. Average scores of SSIM on the test sets of the K562 and CH12-LX cell lines with different down-sampled ratios; Table S6. Average scores of PSNR on the test sets of the K562 and CH12-LX cell lines with different down-sampled ratios; Figure S1. Training performance of HiCENT for MSE, $L_{total}$, PSNR and SSIM as the number epochs increases.
