# Supplementary material for: Enhancing Single-Cell and Bulk Hi-C Data Using a Generative Transformer Model
Source: Biology (Basel). 2025 Mar 12;14(3):288. doi: 10.3390/biology14030288 (PMC11940666; doi:10.3390/biology14030288)

Supplementary Materials

This file includes Table S1-S6 and Figure S1.

Table S1. Summary of Hi-C and scHi-C enhancement methods evaluated in this study. CNN: Convolutional Neural Networks, ResNet: Residual Networks, GAN: Generative Adversarial Networks.

Methods	Category	Data	Source code
HiCENT	CNN, Transformer	Hi-C/scHi-C	https://github.com/shaoqiangzhang/HiCENT
HiCPlus	CNN	Hi-C	https://github.com/zhangyan32/HiCPlus_pytorch
DeepHiC	GAN	Hi-C	https://github.com/omegahh/DeepHiC
HiCNN	CNN	Hi-C	http://dna.cs.miami.edu/HiCNN/
HiCSR	Autoencoder, GAN	Hi-C	https://github.com/PSI-Lab/HiCSR
HiCARN	ResNet, GAN	Hi-C	https://github.com/OluwadareLab/HiCARN
ScHiCEDRN	ResNet, GAN	scHi-C	https://github.com/BioinfoMachineLearning/ScHiCEDRN

Table S2. The components and parameters of all models. (G: generators, D: discriminators, DAE: denoising autoencoder, CL: Convolutional Layers, AF: Activation Function, TE: Training Epochs).

Model	Architecture	Channels	CL	Heads	AF	TE
HiCENT	CNN+Transformer	32	114	8	ReLU, SwiGLU	20
HiCPlus	CNN	64	3	0	ReLU	1350
DeepHiC	GAN	64	20 (G:13+D:7)	0	G: tanh,Sigmoid<br>D: Sigmoid	200
HiCNN	CNN	64	137	0	ReLU	200
HiCSR	GAN	64	47<br>(G:37+D:5+DAE:5)	0	G: ReLU<br>D: Leaky ReLU,<br>DAE: ReLU	250
HiCARN	GAN	64	41 (G:32 + D:9)	0	G: ReLU<br>D: Leaky ReLU,<br>Sigmoid	100
ScHiCEDRN	GAN	64	39 (G:66 + D:9)	0	G: ReLU<br>D: Sigmoid	100

Table S3. Training times and memory usage of each competing model on GM12878. All models were individually trained using an nVidia GeForce RTX 4090 with 24Gb of memory.

Model	Training time(hours)	Memory
HiCENT	1.96	15.72%
HiCPlus	2.11	6.24%
DeepHiC	5.34	13.95%
HiCNN	24.78	40.67%
HiCSR	19.93	24.26%
HiCARN	5.82	23.14%

Table S4. Average scores of GenomeDISCO on the test sets of the K562 and CH12-LX cell lines with different down-sampled ratios.

Cell line	Down-sampled ratio	HiCPlus	DeepHiC	HiCNN	HiCSR	HiCARN	HiCENT
K562	1/16	0.8259	0.8392	0.8290	0.6586	0.8509	**0.8588**
	1/32	0.8303	0.8424	0.8196	0.7915	0.8536	**0.8550**
	1/64	0.8235	0.8235	0.8350	0.8156	0.8455	**0.8472**
	1/100	0.8113	0.8225	0.8352	0.8363	0.8358	**0.8379**
CH12-LX	1/16	0.6157	0.6126	0.6001	0.5174	0.604	**0.6332**
	1/32	0.6052	0.6113	0.5995	0.4622	0.6164	**0.6293**
	1/64	0.6038	0.5965	0.6097	0.4183	0.6188	**0.6250**
	1/100	0.5898	0.5794	0.6014	0.6053	0.6012	**0.6129**

Table S5. Average scores of SSIM on the test sets of the K562 and CH12-LX cell lines with different down-sampled ratios.

Cell line	Down-sampled ratio	HiCPlus	DeepHiC	HiCNN	HiCSR	HiCARN	HiCENT
K562	1/16	0.9124	0.9203	0.9286	0.9226	0.9378	**0.9428**
	1/32	0.8065	0.7845	0.8269	0.8524	0.8214	**0.8368**
	1/64	0.5184	0.5833	0.5873	0.5911	0.5642	**0.6026**
	1/100	0.3915	0.4003	0.3772	0.3941	0.4177	**0.4217**
CH12-LX	1/16	0.9329	0.9405	0.9522	0.9111	0.9699	**0.9727**
	1/32	0.8813	0.8678	0.8844	0.8982	0.9059	**0.9152**
	1/64	0.6734	0.7829	0.7417	0.7716	0.7508	**0.7832**
	1/100	0.5879	**0.6528**	0.5543	0.5672	0.6443	0.6446

Table S6. Average scores of PSNR on the test sets of the K562 and CH12-LX cell lines with different down-sampled ratios.

Cell line	Down-sampled ratio	HiCPlus	DeepHiC	HiCNN	HiCSR	HiCARN	HiCENT
K562	1/16	30.7121	33.6599	33.0259	32.9530	34.1962	**35.0863**
	1/32	25.1642	26.1034	**26.5667**	25.8766	26.1452	26.0413
	1/64	19.5623	19.5922	19.5712	19.3615	19.5622	**19.7509**
	1/100	16.4245	16.2297	15.8765	**17.0644**	16.4017	16.521
CH12-LX	1/16	28.5488	34.2514	32.5746	31.9129	35.4234	**35.9764**
	1/32	24.6425	28.4166	26.5828	26.0536	**28.7029**	28.4349
	1/64	24.0605	23.9696	21.9503	23.2623	24.1735	**24.1963**
	1/100	**23.0177**	21.8055	21.5174	21.7414	21.6943	21.7316

Figure S1. Training performance of HiCENT for MSE, L_{total}, PSNR and SSIM as the number epochs increases. Each epoch consists of 1,000 batch samples.

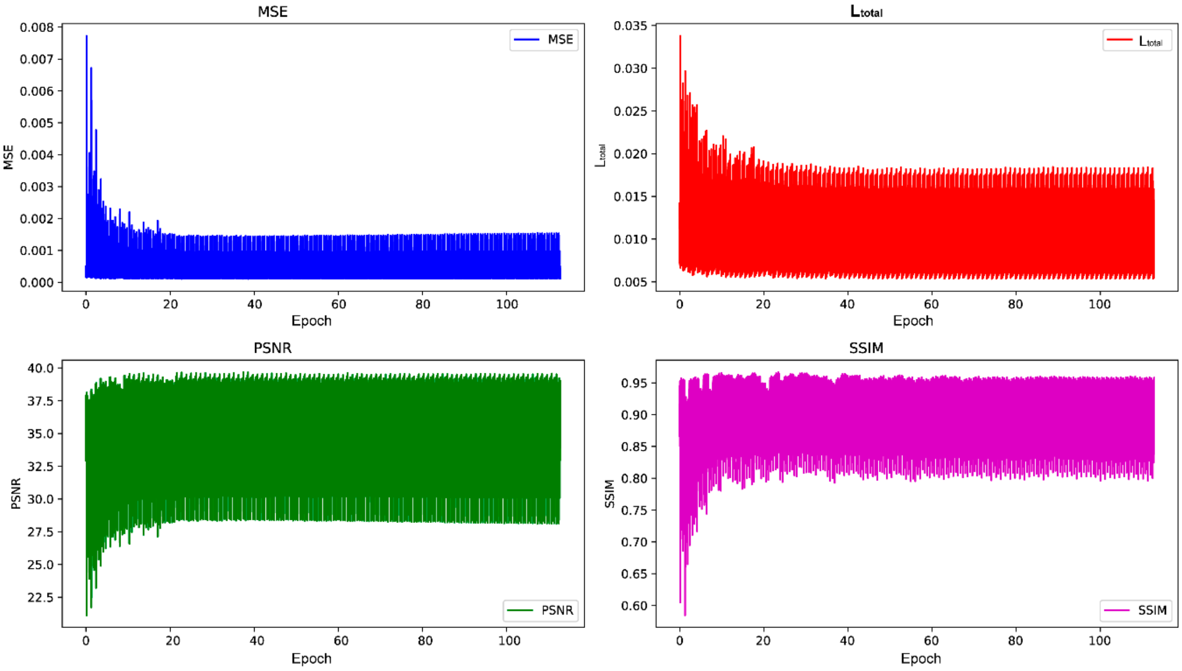

Supplement: Supplementary file 1 [file biology-14-00288-s001.zip › biology-3499094-supplementary.pdf]
